# Generation and characterization of novel anti-DR4 and anti-DR5 antibodies developed by genetic immunization

**DOI:** 10.1038/s41419-019-1343-5

**Published:** 2019-02-04

**Authors:** Agathe Dubuisson, Cécile Favreau, Eric Fourmaux, Sabrina Lareure, Rafael Rodrigues-Saraiva, Catherine Pellat-Deceunynck, Said El Alaoui, Olivier Micheau

**Affiliations:** 1 Université Bourgogne Franche-Comté, INSERM, LNC UMR1231, F-21079 Dijon, France; 2 Research Department, CovalAb, 11 Avenue Albert Einstein, 69100 Villeurbanne, Lyon, France; 3 INSERM, UMR1231, « Equipe labellisée Ligue contre le Cancer » and Laboratoire d’Excellence LipSTIC, F-21079 Dijon, France; 4 CRCINA, INSERM, CNRS, Université d’Angers, Université de Nantes, Nantes, France

## Abstract

Development of therapeutic antibodies in oncology has attracted much interest in the past decades. More than 30 of them have been approved and are being used to treat patients suffering from cancer. Despite encouraging results, and albeit most clinical trials aiming at evaluating monoclonal antibodies directed against TRAIL agonist receptors have been discontinued, DR4 or DR5 remain interesting targets, since these receptors are overexpressed by tumour cells and are able to trigger their death. In an effort to develop novel and specific anti-DR4 and anti-DR5 antibodies with improved properties, we used genetic immunization to express native proteins in vivo. Injection of DR4 and DR5 cDNA into the tail veins of mice elicited significant humoral anti-DR4 and anti-DR5 responses and fusions of the corresponding spleens resulted in numerous hybridomas secreting antibodies that could specifically recognize DR4 or DR5 in their native forms. All antibodies bound specifically to their targets with a very high affinity, from picomolar to nanomolar range. Among the 21 anti-DR4 and anti-DR5 monoclonal antibodies that we have produced and purified, two displayed proapoptotic properties alone, five induced apoptosis after cross-linking, four were found to potentiate TRAIL-induced apoptosis and three displayed antiapoptotic potential. The most potent anti-DR4 antibody, C#16, was assessed in vivo and was found, alone, to inhibit tumour growth in animal models. This is the first demonstration that DNA-based immunization method can be used to generate novel monoclonal antibodies targeting receptors of the TNF superfamily that may constitute new therapeutic agents.

## Introduction

TRAIL (tumour necrosis factor-related apoptosis inducing ligand) agonist receptors, DR4 and DR5, have for more than two decades been considered as potential targets for cancer therapy owing to their ability to trigger selective apoptosis in tumour cells while sparing normal cells^1,2^. Clinical evaluations of TRAIL, the natural ligand of DR4 and DR5, or anti-DR4/DR5 antibodies, alone or combined with chemotherapy, have, however, been discontinued owing to lack of efficacy^3^. The sole exceptions, to date, include the clinical studies of the novel TRAIL recombinant protein developed by Sunbio Biotech^4^. The recombinant protein Circularly Permuted TRAIL has been found to increase objective response in patients suffering from multiple myeloma, alone or combined with thalidomide^5,6^. Engagement of apoptosis by TRAIL agonist receptors mostly relies on the ability of the ligand or agonist antibodies to induce receptor aggregation. Accordingly, increasing TRAIL agonist valency enhances their proapoptotic activity up to 100-fold^7–9^. Mechanistically, the binding of TRAIL or agonist antibodies to DR4 and DR5 allows recruitment of the adaptor protein FADD, which in turn enables the binding of the initiator cysteine protease, caspase-8, within a supramolecular scaffold coined DISC for Death Inducing Signalling Complex^10^. Within this scaffold, the zymogen caspase gets activated by proximity^11^ and released to the cytosol, where it can cleave other proteases such as caspase-3, another cysteine protease responsible for the execution phase of the apoptotic machinery. To date, antibodies assessed in the clinic have mostly focussed on DR5, with only one anti-DR4 evaluated in phase I/II^4^. Keeping in mind that DR4 prevails over DR5 in transducing TRAIL-induced cell death^12^ or that anti-DR5 and anti-DR4 antibodies can potentiate TRAIL-induced cell death^13,14^, development of antibodies targeting these receptors still holds interest in oncology.

Development of therapeutic antibodies by immunization is today an optimized and well-controlled process. Conventional immunization is mostly based on recombinant proteins or peptides^15–17^. However, not all targets can be reproduced recombinantly. Their highly hydrophobic nature or conformational and topological complexity can make them difficult to produce^18^. In particular, generation of antibodies directed against multiple transmembrane proteins has proved to be difficult. Moreover, protein folding can be altered by the production and purification processes or due to the lack of their transmembrane domain. The difficulty to generate certain posttranslational modifications makes the endogenous and native conformations of the immunogenic protein and its native epitopes hard to reproduce in vitro^19,20^. To overcome these problems, a new approach of immunization was developed in the early 90s, coined genetic immunization^21,22^. Originally developed as a vaccination method^23^, DNA immunization has demonstrated its ability to induce significant cellular and humoral response^24^. DNA immunization was found to be more effective than protein immunization in activating B cells in the germinal centre. It has also presented advantages for the production of monoclonal antibodies (mAbs)^25^. We describe here the characterization of several monoclonal anti-DR4 and -DR5 antibodies developed by this approach displaying proapoptotic activity or able to synergize with TRAIL.

## Materials and methods

### Ligand, chemicals and antibodies

His-tagged human TRAIL was produced and used as described previously^26^. The anti-DR4 (clone wB-K32) and anti-DR5 (clone B-L27) antibodies, from Diaclone (Besançon, France), were used for flow cytometry^27^. Alexa-488-conjugated-goat anti-mouse secondary antibody was from Molecular Probes (Life Technologies, Saint Aubin, France). For apoptosis measurement, Annexin V (No. 556422) and 7-aminoactinomycin D (7AAD; No. 559925) were from BD Biosciences (Le Pont de Claix, France). For western blot analysis, anti-DR4 (clone AB16955) antibody was purchased from Chemicon (Millipore, Molsheim, France). Antibodies against caspase-3 (clone MF393), caspase-8 (clone 5F7) and anti-caspase-9 (clone 5B4) were from Medical & Biological Laboratories (Clinisciences, Montrouge, France). The anti-cleaved poly ADP-ribose polymerase-1 (PARP-1; clone D64-E10) was from Cell Signaling Technology (Ozyme, Saint Quentin, France). Antibodies against PARP-1 (clone H-300), GAPDH (clone 0411) and HSC70 (clone B-6) were from Santa Cruz Biotechnology (CliniSciences). Horseradish peroxidase (HRP)-conjugated anti-rabbit was from Jackson ImmunoResearch (Interchim, Montluçon, France). HRP-conjugated anti-mouse immunoglobulin heavy chain+light chain (IgG (H+L)) secondary antibody was from CovalAb (Villeurbanne, France). HRP-conjugated anti-mouse IgG1-, Ig2a- and Ig2b-specific antibodies were obtained from Southern Biotech (Clinisciences). The caspase inhibitor, Q-VD-Oph, was from Selleck Chemicals (Munich, Germany). Puromycin was purchased from InvivoGen (Toulouse, France); IdeZ enzyme from Promega (Charbonnières, France). Protein A high-performance affinity columns (cat# 17-0402-01) and NPROTEIN A SEPH 4 FF (cat# 17-5280-02) were from GE Healthcare (Dutscher, Brumath, France). Hypoxanthine–aminopterin–thymidine (HAT; cat# H0262) and polyethyl glycol (PEG; cat# P2906) were from Sigma (St Quentin Fallavier, France). Hhybridoma cloning supplement (HCS; cat# sc-224479) from Santa Cruz.

### Animals

Mice used for these studies were maintained in a specific pathogen-free zone in accredited animal facility. All experiments were performed in compliance with the Committee of Ethics of Grand Campus de Dijon (C2EA grand campus Dijon) no. 105 – C2EA105. Eight-week-old female Balb/c mice (Charles Rivers France, St Aubin-les-Elbeuf) were used for the immunization protocol, and 8-week-old female Balb/c nude mice (Charles Rivers) were used for the tumour growth evaluation following antibody injections.

### Plasmid constructs

Plasmids encoding soluble human recombinant versions of DR4 or DR5 fused to the constant fraction of human gamma-immunoglobulin, i.e. DR4 (DR4-Fc) or DR5 (DR5-Fc), and conferring resistance to puromycin were constructed by PCR following In-Fusion manufacturer’s instructions (In-Fusion HD cloning, Takara, Clontech), using the following primers OR435 5’-GCG-AAA-CGA-TCC-TCA-TCC-TGT-CTC-TTG-ATC-GATC-3’; OR436 5’-TTT-CGA-TAA-GCC-AGT-AAG-CAG-TGG-GTT-CTC-TAG-3’; OR437 5’-ACT-GGC-TTA-TCG-AAA-TTA-ATA-CGA-CTC-ACT-ATA-GGG-AGA-CCC-AAG-3’; OR438 5’-GTG-CCT-GAG-CGG-GAC-TCT-GGG-GTT-3’; OR439 5’-TGA-GGA-TCG-TTT-CGC-CAT-GAC-CGA-GTA-CAA-GCC-C-3’ and OR446 5’-GTC-CCG-CTC-AGG-CAC-CGG-GCT-TGC-3’. Briefly, the neomycin cassette of the pCR3-neo-hDR4-Fc and pCR3-neo-hDR5-Fc expression vectors (generous gift from Dr Pascal Schneider, Epalinges, Switzerland) was replaced by puromycin by assembling PCR fragments obtained using OR435/OR436 and OR439/OR446 from each vector to the cDNA encoding resistance to puromycin generated by PCR amplification from OM181, a retroviral vector derived from pMSCV-Puro, resulting in the pCR3-Puro-hDR4-Fc (OM1450) and pCR3-Puro-hDR5-Fc (OM1449) constructs.

The pCR3-based plasmids encoding DR4 or DR5 full length (pCR3-DR4 and pCR3-DR5) under the control of the CMV promoter were kindly given by Dr Pascal Schneider. The CAG promoter-derivative plasmid encoding WPRE and GM-CSF full length, pCAGGS-GM-CSF (OM1463), was obtained by PCR, using the pCAGGS plasmid described by Niwa et al.^28^, a WPRE containing vector synthesized from Genscript (OM567), a plasmid encoding mGM-CSF cDNA obtained from Riken (RDB 1687, OM1438) and a bGH Poly(A) signal containing vector obtained from genscript (GenEZ ORF clone OMU22984D, OM1459) and the following primers were used: OR504 5’-CGC-CTC-CCC-GCC-CTG-TGC-CTT-CTA-GTT-GCC-AGC-3’; OR505 5’-GGC-TTC-ATG-ATG-TCC-CCA-TAA-TTT-TTG-GCA-GAG-GGA-AAA-AGA-TCT-CCA-TAG-AGC-CCA-CCG-CAT-C-3’; OR506 5’-AAA-CCA-AGC-CAA-AAA-TGA-GAA-TTC-GAA-TCA-ACC-TCT-GGA-TTA-CAA-AAT-TTG-TGA-AAG-A-3’; OR507 5’-AGG-GGC-AAA-CAA-CAG-ATG-GCT-GGC-AAC-TAG-AAG-GCA-CAG-GGC-GGG-GAG-GCG-3’; OR 510 5’-AAC-GTG-CTG-GTT-ATT-GTG-CTG-TCT-CAT-CAT-TTT-GGC-AAA-GAA-TTC-AAA-TGT-GGC-TGC-AGA-ATT-TAC-TTT-TCC-T-3’; OR511 5’-AAT-TTT-GTA-ATC-CAG-AGG-TTG-ATT-CGA-ATT-CTC-ATT-TTT-GGC-TTG-GTT-TTT-TGC-ATT-CAA-AGG-GGA-3’. Briefly, pCAGGS was digested using EcoRI and BglII and ligated using the In-Fusion method with PCR products amplified from OM1459 using OR504/505, OM567 using OR506/507 and OM1438 using OR510/511. The CAG promoter plasmid encoding WPRE and DR5 full length (pCAGGS-DR5) was obtained by digesting OM1463 with EcoRI, to replace mGM-CSF by the full-length sequence of DR5, as above using the PCR fragments obtained from the pCR3-DR5 plasmid using OR570 5’-ATC-ATT-TTG-GCA-AAG-AAT-TCC-CAT-GGA-ACA-ACG-GGG-ACA-GAA-CG-3’ and OR571 5’-AAT-CCA-GAG-GTT-GAT-TCG-AAT-TCA-GGA-GGA-CAT-GGC-AGA-GTC-TGC-A-3’. After bacterial transformation, all final constructs were confirmed by sequencing. When sequencing had been confirmed, LPS-Free MAXI plasmid preparations were performed (Macherey-Nagel, NucleoBond® Xtra Maxi EF).

### Cell lines

293T cells and the colorectal and breast cancer cell lines, HCT-116 and MDA-MB-231, respectively, were cultured in Dulbecco’s minimum essential medium (DMEM; Lonza, Levallois-Perret, France) supplemented with 10% of foetal calf serum (FCS; Lonza). 293T expressing stably hDR4-Fc and hDR5-Fc (described below) and MDA-MB-231 or HCT116 isogenic derivatives, deficient for TRAIL receptors (DKO), and DKO reconstituted with DR4 (DKO-DR4^rec^) and DR5 (DKO-DR5^rec^)^12,29^ were cultured as above in DMEM. The lung carcinoma cell line H1703 was cultured in RPMI medium (Lonza) completed with 10% of FCS. For cell maintenance, adherent cells were washed with HBSS (Lonza), detached with trypsin and diluted 1/10th with complete medium three times a week. The Burkitt Lymphoma cell line BL2 was cultured in RPMI medium completed with 10% of FCS.

### Recombinant protein production

DR4-Fc and DR5-Fc expressing stable cell lines (293T-DR4-Fc and 293T-DR5-Fc, respectively) were obtained by transfections of the pCR3-DRs-Fc plasmids (OM1449 or OM1450) into 293T cells cultured in a 10-cm tissue culture dish at 60% confluence. Transfections were performed using the Lipofectamine 3000, according to the provider’s recommendations (Thermo Fisher Scientific, Courtaboeuf, France). Transfected cells were grown in culture medium for 24 h, then treated with DMEM supplemented with 2.5 µg/mL of Puromycin. After selection, 293T-DR4-Fc and 293T-DR5-Fc cells were cultured in T175-mm^2^ culture flasks. When the confluence reached 80%, the medium was changed to a synthetic medium, OptiMEM Glutamax (Thermo Fisher Scientific), to boost protein production. After 5 days of incubation, the supernatant was harvested and proteins were purified as follows. Supernatant containing recombinant DRs-Fc protein was passed onto a high-performance protein-A column, washed twice with cold phosphate-buffered saline (PBS) 1×, then recombinant proteins were eluted with a 0.1 M citrate NaOH pH 2.5 solution, buffered with a 1 M Tris-HCl pH 9. The resulting pH 7 neutral solution was sterilized using apyrogenic 0.2-micron filtration membranes and concentrated with Centricon (Millipore) to reach the concentration of 1 or 2 mg/mL. Cleaved receptors, devoid of Fc, cDR4 and cDR5 recombinant proteins were obtained using IdeZ enzyme following the manufacturer’s recommendations.

### DNA immunization protocol

Balb/c mice were immunized with either 50 µg pCR3-DR4 or 50 µg pCAGGS-DR5 and 2.5 µg pCAGGS-GMCSF diluted in saline buffer via hydrodynamic tail vein (HTV) injection as previously described^30^. DR4 DNA injections were performed once a week during 4 weeks, whereas DR5 DNA injections were performed every 14 days during 8 weeks. To follow-up the production of antibodies, blood samples were taken after the third HTV immunization and serums were tested by enzyme-linked immunosorbent assay (ELISA).

### Detection of human anti-DR antibody titration by ELISA

Determination of anti-DR4 or anti-DR5 antibody titres from mice sera, hybridoma supernatants or purified antibodies was assessed by ELISA. Sera from Balb/c mice were taken during immunization, hybridoma supernatants and purified antibodies were tested during mAb development. They were screened on recombinant cleaved soluble receptors, cDR4 and cDR5, respectively. ELISA have been performed as follows. Recombinant cDR4 and cDR5 were coated to 96-well EIA/RIA flat bottom microtitre plates (Corning, Costar 3590, Thermo Fisher Scientific). Hybridoma supernatant samples were diluted by half; sera and purified antibodies were serially diluted by half. After binding for 1 h at 37 °C on a rotating plate and incubation with the secondary antibody, reactivity was measured at 450 nm using a microplate reader (Labsystems Multiskan Ascent, Bradenton, USA). Samples were considered positive when the optical density was >0.3 for supernatant of hybridomas. Titres of purified antibodies were evaluated as the highest dilution (in ng/mL) where optical density is ≥1.

### Production of mAbs

Fusions were performed from the most responsive mice. Briefly, spleens were harvested and splenocytes were extracted then fused to Sp2/0Ag mouse myeloma cells using PEG^31^. In all, 2 × 10^5^ cells/mL were then distributed into 96-well plate with medium containing 20% FCS, HAT and HCS. Three weeks post-fusion, supernatants were screened for antibody production by ELISA. All antibody-secreting hybridomas demonstrating significant positivity by flow cytometry underwent two rounds of sub-cloning. Selected sub-clones were then expanded for large-scale production. Twenty-one clonal hybridoma supernatants were then purified by Protein-A affinity chromatography.

### Flow cytometry

Determination of selectivity was performed by flow cytometry using HCT116 cells or isogenic cell lines originating from MDA-MB-231 cells, expressing either DR4 or DR5 or none (DKO) of these receptors^12^. Flow cytometric analyses were performed using either hybridoma supernatants or purified antibodies. Hybridoma supernatants were screened on parental HCT116 and HCT116-DKO cells for DR4 clones and on MDA-MB-231 DKO and isogenic MDA-MB-231-DKO-DR5^rec^ cells for DR5 clones. Purified antibodies were screened on MDA-MB-231-DKO, MDA-MB-231-DKO-DR4^rec^ and MDA-MB-231-DKO-DR5^rec^ cells. Cells were collected from culture flasks, washed with ice-cold PBS (Sigma) and dispatched in FACS tubes at 10^5^ cells per condition. Hybridoma supernatants were screened neat, and purified antibodies were diluted 1/100th in PBS containing 3% FCS. Samples were added to cells and incubated at 4 °C for 30–60 min. Tubes were then centrifuged and washed with PBS. Cells were then incubated at 4 °C for 30–40 min with AF488-conjugated goat anti-mouse IgG (H+L) diluted 1/200th in PBS containing 3% FCS and washed prior analysis by flow cytometry using a FACS-CANTO flow cytometer (BD Biosciences). Analysis were next performed using the FlowJo software.

### Affinity determination

The affinity and cross-reactivity were determined using an Octet Red system and anti-human IgG quantification (AHQ) biosensors (FortéBIO, St Germain en Laye, France). All reagents were prepared in binding buffer (TRIS-NaOH 0.1 M, pH 7.4). Recombinant DR4-Fc and DR5-Fc were prepared at 5 µg/mL in binding buffer, fixed to the biosensor and then put in contact with the antibody of interest. The assay plate was agitated at 1000 rpm at 30 °C. Two columns (8 each) of biosensors were pre-hydrated in binding buffer for 10 min. Anti-hIgG biosensors were baselined for 180 s before and after loading of recombinant DR4-Fc or DR5-Fc (800 s). The binding kinetics were measured by dipping loaded biosensors (120 s) in varying concentrations of mAbs (from 500 nM to 15 nM). Next, dissociation was recorded for an additional 600 s. Interactions were monitored during association and dissociation period. Biosensors were then regenerated with three cycles of 5 s in regeneration buffer (citrate-NaOH pH 2.5) followed by neutralization in binding buffer. The data were fit to a 1:1 binding stoichiometric model. All octet experiments were designed and analysed with the FortéBio data acquisition software (7.1) and Data Analysis Software version 7.1.0.36 with Savitsky–Golay filtering to reduce noise. The data were adjusted with the version 5 of the GraphPad software.

### Viability and apoptosis assays

Effects of mAbs of interest were measured on HCT116, MDA-MB-231 and BL2 cells. Viability was determined using methylene blue assay as described earlier^30^. HCT116 or MDA-MB-231 (5 × 10^5^/mL cells) were plated into 96-well plates and incubated overnight at 37 °C with cascade dilutions of treatments (mAbs alone, TRAIL alone and an isoconcentrations of combined mAbs and TRAIL) starting from 10 µg/mL. Supernatants containing or not dead cells were discarded, wells were washed with PBS and remaining adherent cells were fixed with methanol. After a 15-min incubation, methanol was discarded, dried and cells were stained using methylene blue for 15 min at room temperature. Stained cells were extensively washed, and plates were dried for 2 h at 37 °C. Subsequently, methylene blue was eluted with HCl 0.1 M and absorbance was measured at 630 nm using the Asys UVM 340 microplate reader from Biochrom (Cambridge, UK). Percentage of cell viability vs. medium was calculated relative to non-stimulated cells, corresponding to 100% survival.

Apoptosis induced by mAbs combined or not to TRAIL was quantified by allophycocyanin Annexin V (fluorescein isothiocyanate) and 7AAD staining, according to the manufacturer’s instructions (BD Biosciences), and analysed by flow cytometry (FACS LSRII). BL2 cells were plated at 2 × 10^5^ cells/mL in 24-well plates and incubated with mAbs at 10 µg/mL alone or in combination with 1 µg/mL TRAIL for 20 h. Prior treatments, corresponding wells were treated with 10 µM of Q-VD-Oph (Selleckchem), a caspase inhibitor, for 30 min at 37 °C. MDA-MB-231 cells were plated at 1 × 10^6^ cells/mL in T25-mm^2^ flasks and incubated with mAbs at 5 µg/mL alone or combined to 1 µg/mL TRAIL for 8 h. The inhibitory apoptosis potential of C#21 and C#23 antibodies was assessed by flow cytometry on HCT116-DKO-DR5^rec^ as described above. To test the activity of C#16, in human cancer cell lines, BL2, H1703, HCT116 and MDA-MB-231, cells were plated at 2 × 10^5^ cells/mL in 24-well plates and incubated with a dilution of C#16 ranging from 10 to 0.3 µg/mL for 24 h. All the above-mentioned experiments were repeated at least three times.

### Immunoblot analyses

Dot blots were performed using purified recombinant DR4 and DR5. Soluble receptors, denatured or not in the presence of 1 M dithiothreitol (DTT) for 5 min at 95 °C, were deposited onto nitrocellulose membranes and dried for 15 min. Membranes were then blocked in PBS-T (PBS, 0.5% Tween 20) containing 5% non-fat dry milk (PBS-TM) for 1 h and incubated with anti-DR4 and anti-DR5 antibodies diluted 1/1000th overnight at 4 °C. The next day, the membranes were washed three times with PBS-T and the binding was detected with an anti-mouse IgG (H + L)–HRP antibody.

For immunoblots, BL2, H1703, HCT116 or MDA-MB-231 cells were put at 1 × 10^6^ cells/mL in T25-mm^2^ flasks and incubated with mAbs at 5 or 1 µg/mL alone or in combination with 1 µg/mL of TRAIL for a final volume of 10 mL. Supernatants and cells were collected 8 h later and cell lysates were prepared using NP40 lysis buffer containing 1% NP40, 20 mM Tris-HCl pH 7.5, 150 mM NaCl, 10% glycerol and a proteinase inhibitor cocktail (Roche, Meylan, France). Cell extracts were run on 12% sodium dodecyl sulfate-polyacrylamide gel electrophoresis gels. Proteins were transferred to nitrocellulose membrane (Bio-Rad) by liquid electro-blotting using the criterion blotter apparatus (Bio-Rad). Membranes were next saturated using PBS-TM for 1 h and incubated with primary antibodies in PBS-TM overnight at 4 °C on a rotating machine. Washed three times with PBS-T before incubation for 1 h with HRP-conjugated secondary antibodies diluted 1/10,000th in PBS-TM, membranes were then washed three times with PBS-T and immunoreactivity detected using the chemiluminescence detection kit Western BrightQuantum Kit from Advansta (Menlo Park, CA, USA).

### In vivo assays

Female athymic nude mice (Balb/c nu/nu) aged between 7 and 8 weeks were injected subcutaneously in the lower right flank with 1 × 10^7^ BL2 cells mixed with Matrigel (Corning). After randomization, 5–6 mice were injected i.p. with C#16 (10 mg/kg) or vehicle control (PBS). For pre-established xenograft tumour model, when tumour reached approximately 100 mm^3^, mice were injected with C#16 (10 mg/kg) or vehicle control (PBS) on days 16, 20, 24, 28 and 32 post BL2 inoculation. For de novo model, mice were injected with C#16 (10 mg/kg) or vehicle control (PBS) on days 4, 8, 12, 16, 20 and 24 post BL2 inoculation. For all groups, tumour size was measured on two axes with digital callipers every 2 days. The values were transformed into tumour volumes using the following formula: tumour volume (mm^3^) = (Length × Width^2^)/2. Mice were euthanized when the tumour reached 1500 mm^3^ or at the end of the experiment (day 34).

### Statistical analysis

Unless specified, statistical analyses were conducted with the GraphPad software by comparing two group by paired *t* test and all the groups with each other using analysis of variance and Bonferroni’s post-test. For all tests, only data resulting in *p* values <0.05 were regarded as statistically significant.

## Results

### Generation of anti-DR4 and -DR5 antibodies by DNA immunization

DNA immunizations were performed using a proprietary CovalAb protocol, based on previous studies^32^. Mice were immunized with DR4 or DR5 plasmids and immune reactivity was evaluated by ELISA on mice serum. When a good titre was obtained, spleens of selected mice were fused to obtain hybridoma cells. After 3 weeks of culture, the supernatants from the resulting hybridomas were screened by ELISA (Figure S1A). Out of this screening, several antibody-secreting clones were analysed by dot blot and FACS analysis to determine their potential ability to bind to recombinant DR4 and/or DR5 or native receptors on parental HCT116 or isogenic MDA-MB-231 cells expressing solely DR5^12^, as indicated. All antibodies targeting DR4 (Figure S1B) or DR5 (Figure S1C) exhibited positive signal for their corresponding targets. None of the above-described antibodies bound MDA-MB-231-DKO cells deficient for DR4 and DR5 (Figure S1C and not shown). Of these, a limited number of them was next chosen for antibody production and purification.

### Characterization of purified anti-DR4 and anti-DR5 antibodies by ELISA, dot blot and flow cytometry

**Fig. 1:**
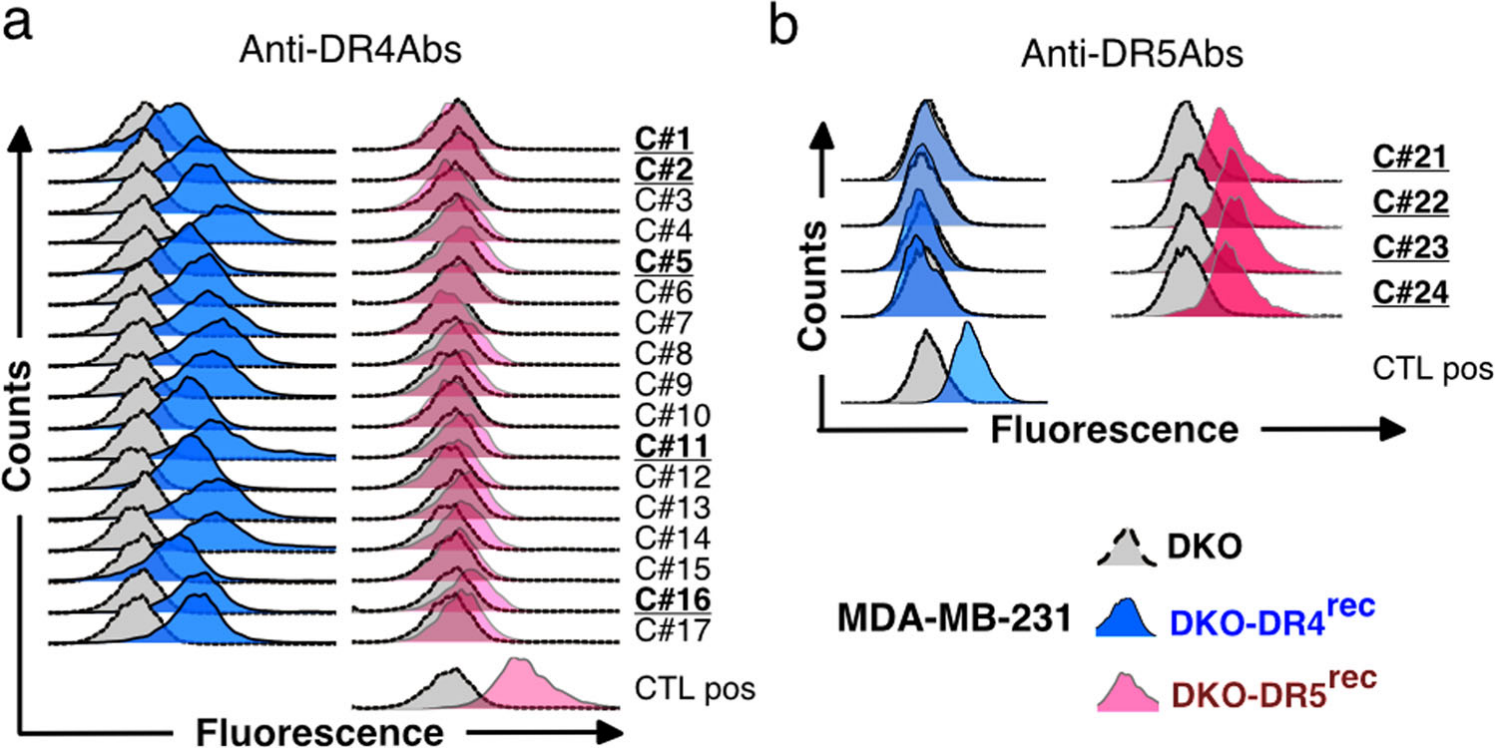
Cell surface receptor recognition of the produced anti-DR4 and anti-DR5 antibodies. MDA-MB-231 DKO (grey curve) and MDA-MB-231 DKO reconstituted with either DR4 (MDA-MB-231-DKO-DR4^rec^, blue curve) or DR5 (MDA-MB-231-DKO-DR5^rec^, red curve) were stained with **a** anti-DR4 antibodies or **b** anti-DR5 antibodies. Primary antibodies were then detected using a AF488-conjugated mouse-specific IgG (H+L) secondary antibody and analysed by flow cytometry

As demonstrated by ELISA, 19 hybridomas produced antibodies displaying good to excellent immune-reactivity with titres ranging from 4 to 80 ng/mL (Figure S2A). Dot blot analysis performed using recombinant cDR4 or cDR5, denatured or not with DTT, indicated that all antibodies generated by the DNA approach recognized specifically the native form of the protein target (Figure S2B). Consistent with this finding, analysis of their binding selectivity by flow cytometry, using MDA-MB-231-DKO, deficient for both receptors (grey histograms), as compared to isogenic cells reconstituted with either DR4 (MDA-MB-231-DKO-DR4^rec^, blue histograms) or DR5 (MDA-MB-231-DKO-DR5^rec^, red histograms), demonstrated that all these mAbs were highly specific for their corresponding targets, namely DR4 (Fig. 1a) or DR5 (Fig. 1b).

### Analysis of the proapoptotic potential of DR4 and DR5 antibodies

**Fig. 2:**
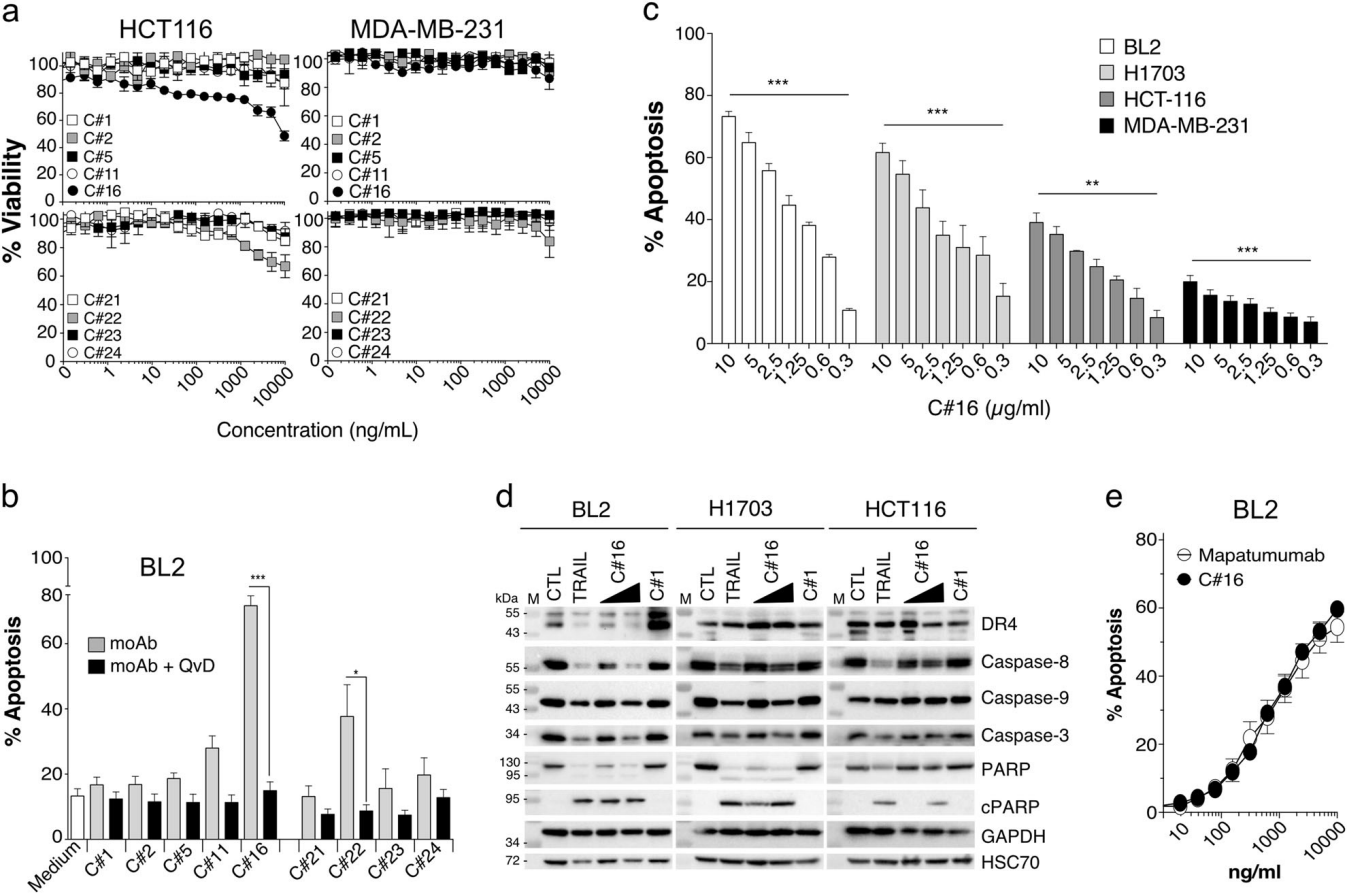
Apoptosis induced by anti-DR4 or -DR5 antibodies. **a** Viability of HCT-116 and MDA-MB-231 were determined using methylene blue, 16 h after treatment with increasing concentrations of anti-DR4 mAbs (C#1, C#2, C#5, C#11 and C#16) or anti-DR5 mAbs (C#21, C#22, C#23 and C#24). Values are means ± SD (*n* = 3). **b** BL2 cells were pre-incubated or not for 30 min at 37 °C with 10 µM of Q-VD-Oph prior stimulation for 20 h with 10 µg/mL of mAbs. Cells were next stained with Annexin V/7AAD and apoptosis was quantified by flow cytometry. **c** BL2, H1703, HCT116 and MDA-MB-231 cell lines were treated for 20 h with increasing concentrations of C#16, and apoptosis was quantified as above by flow cytometry. **d** BL2, H1703 and HCT116 cells were stimulated with 1 µg/mL His-TRAIL, 1 or 5 µg/mL of C#16 or 5 µg/mL C#1 for 8 h. Corresponding cell extracts were analysed by immunoblot. **e** BL2 cells were treated with increasing concentration of C#16 or Mapatumumab for 20 h, and apoptosis was quantified with Annexin V/7AAD staining by flow cytometry. All values are presented here as ±SD (*n* = 3). Significance was evaluated by analysis of variance tests. *p < 0.1, **p < 0.05, ***p < 0.01

**Fig. 3:**
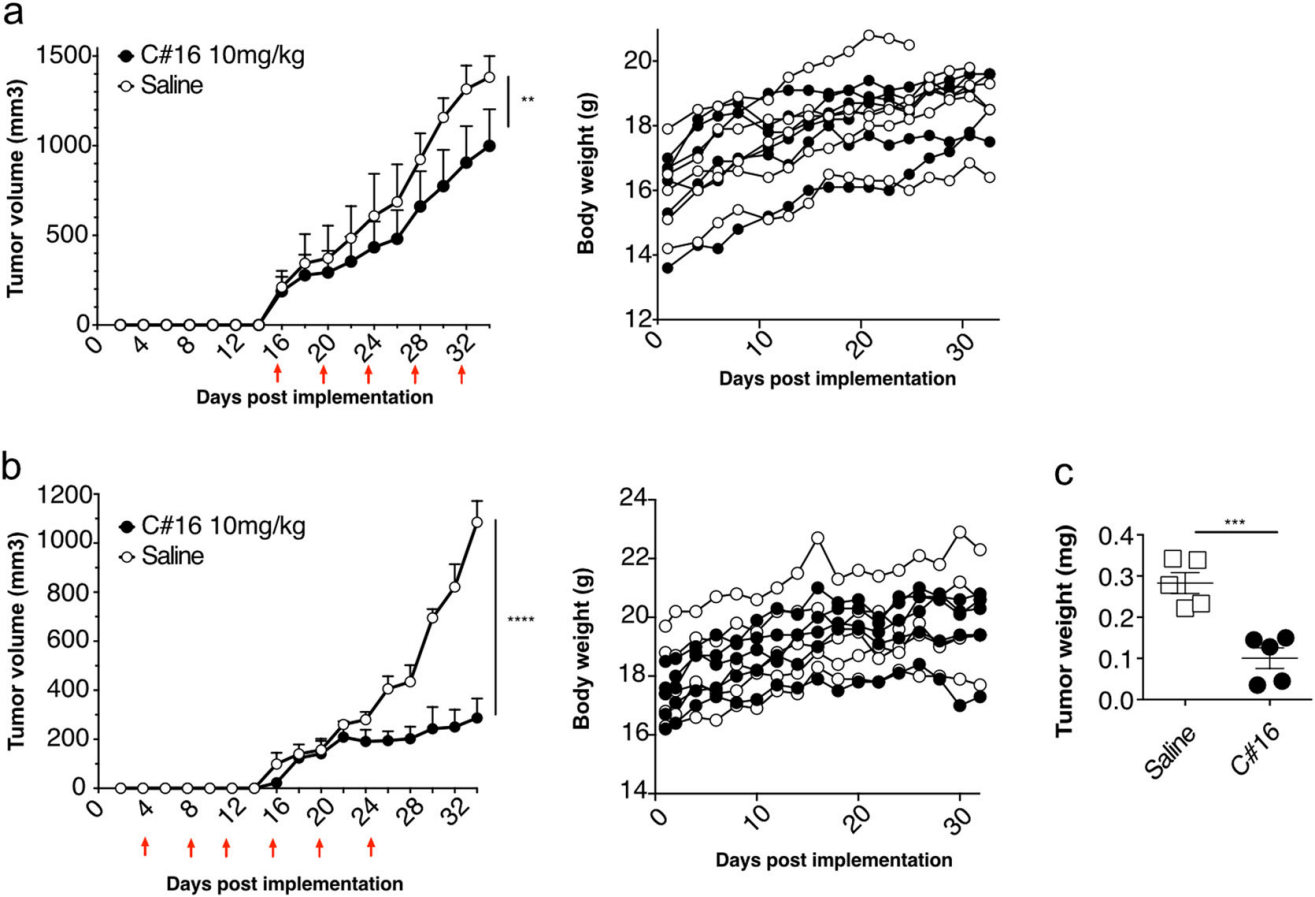
The anti-DR4 antibody C#16 reduces tumour growth in xenograft models. BL2 cells mixed with Matrigel were inoculated into the right flank of mice subcutaneously at day 0. Mice were then randomized into groups of five to six mice and were injected intraperitoneally with C#16 (10 mg/kg) or vehicle control (phosphate-buffered saline) at the indicated times (red arrows). Each time point represents the mean value (±s.e.m) of the tumour sizes on the day of measurement. The animals were sacrificed 34 days postinoculation. **a** Pre-established xenograft tumour growth and corresponding mouse body weight. Significance was evaluated by analysis of variance (ANOVA) test. **p < 0.05. **b** De novo model tumour growth and corresponding mouse body weight. Significance was evaluated by ANOVA test. ****p < 0.0001. **c** Comparison of tumour weights from de novo model between mice injected with saline and mice injected with C#16 (10 mg/kg). ***p < 0.01. Significance was tested using unpaired *t* test

Given that the antibodies recognize the native form of TRAIL agonist receptors, we next investigated whether some of them might display proapoptotic functions. To address this question, five anti-DR4 (clones C#1, C#2, C#5, C#11, C#16) and four anti-DR5 antibodies (clones C#21, C#22, C#23, C#24), were produced in larger quantities and evaluated, by methylene blue or Annexin V staining, for their ability to induce the loss of cell viability or apoptosis in a panel of human tumour cell lines sensitive or resistant to TRAIL-induced cell death^12,33–35^. Remarkably, from this rather limited panel of antibodies, the mAb targeting DR4, C#16, and the anti-DR5, C#22, were both able to partially reduce, alone, the cell viability of the colorectal cancer cell line HCT116, in a dose-dependent manner (Fig. 2a). This finding is particularly remarkable for such a screen, as agonist mAbs developed, so far, against DR4 and DR5, with a few exceptions^36,37^, often require cross-linking agents^13,38–41^ or sensitizing drugs such as the proteasome inhibitor bortezomib^42^ to unmask their proapoptotic potential. None of them, however, appeared to inhibit the cell viability of the TRAIL-resistant breast carcinoma triple-negative cell line MDA-MB-231, albeit, at the highest concentration, C#22 had a tendency to reduce the cell viability of these cells (Fig. 2a). Nonetheless, and consistent with the results obtained in HCT116 cells, C#16 and C#22 were, alone and without cross-linking nor enhancer, able to induce >75% and 35% apoptosis respectively, in BL2 cells (Fig. 2b). As expected, cell death-induced by these two antibodies in BL2 cells was inhibited by the pan-caspase inhibitor Q-VD-Oph (Fig. 2b). Since C#16 is the most potent antibody identified in our screen, its apoptosis-inducing properties were monitored by Annexin V staining in a larger panel of cell lines. Interestingly, C#16 induced apoptosis in a dose-dependent manner not only in HCT116, BL2 and the lung carcinoma H1703 cell lines but also, although to a lesser extent, in MDA-MB-231 cells (Fig. 2c). Consistent with these findings, the immunoblot analysis of caspase activation, caspase substrate cleavage or cleaved products, clearly demonstrated that C#16 through binding to DR4 is able to induce activation of the canonical extrinsic pathway (Fig. 2d). Likewise, disappearance of caspase-8 proform, as well as appearance of cleaved PARP products occurred in BL2, H1703 or HCT116 cells, as efficiently as cells stimulated with TRAIL (Fig. 2d). The anti-DR4 mAb C#1 was used, here, as a negative control, as even at the highest concentration, and contrary to C#16, C#1 was not able to induce the caspase cascade nor the cleavage of endpoint products such as PARP (Fig. 2d). Because C#16 was as efficient as Mapatumumab in inducing apoptosis in BL2 cells (Fig. 2e), its ability to inhibit tumour growth in vivo was assessed in nude mice. For this purpose, BL2 lymphoma cells mixed with Matrigel were implanted in the right flank of nude mice and xenografted animals were treated or not with 5–6 injections of C#16 at 10 mg/kg, every 4 days, either when the tumour reached approximately 100 mm^3^ or 4 days after tumour implantation and randomization. The effect of C#16 in pre-established BL2 tumours was significant, leading to 30% loss of tumour growth (Fig. 3a). When the antibody was injected 4 days after implantation, referred as the de novo experiment, the growth rate of BL2 cells was nearly four-fold lower in mice treated with C#16 (Fig. 3b).

### Both DR4 and DR5 antibodies can synergize with TRAIL to induce apoptosis

**Fig. 4:**
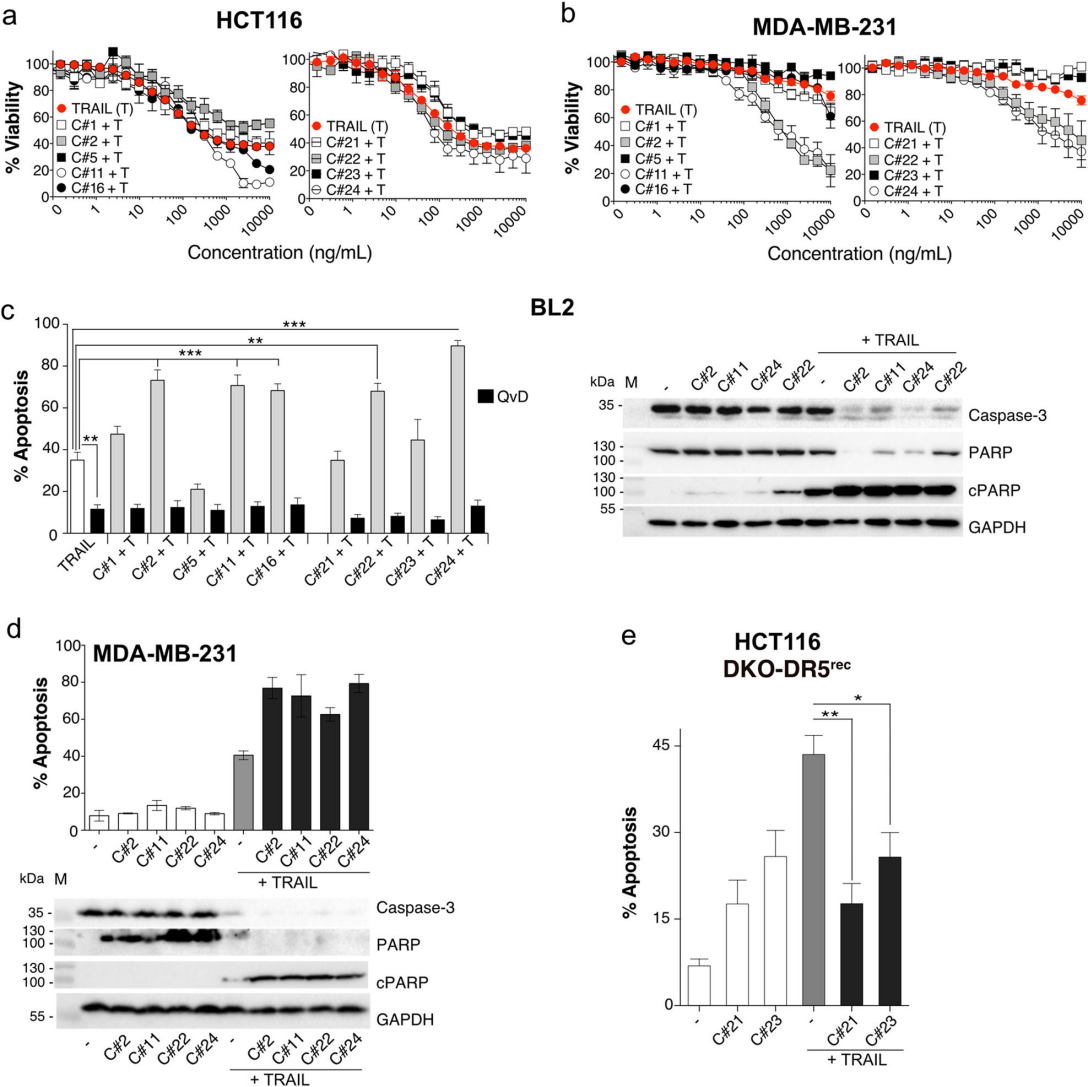
Anti-DR4 or anti-DR5 antibodies can potentiate or inhibit tumour necrosis factor-related apoptosis inducing ligand (TRAIL) action. Cell viability of **a** HCT-116 and **b** MDA-MB-231 cells was determined using methylene blue test. Cells were treated for 16 h with the indicated concentration of DR4 (left) or DR5 (right) monoclonal antibodies (mAbs) in combination with His-TRAIL. Values are means ± SD (*n* = 3). **c** Left: BL2 cells were treated in 24-well plates for 20 h with 1 µg/mL of soluble TRAIL or with a combination of 10 µg/mL of mAbs and 1 µg/mL His-TRAIL. When indicated, 10 µM of Q-VD-Oph was added prior treatment for 30 min at 37 °C. Medium and cells were harvested and apoptosis was detected by Annexin V/7AAD staining and flow cytometry. Significance was evaluated by analysis of variance (ANOVA) test with the mean values ± SD (*n* = 3) **p < 0.05, ***p < 0.01. Right: BL2 were stimulated for 8 h with 5 µg/mL of C#2, C#11, C#22 or C#24 in the presence or absence of 1 µg/mL His-TRAIL and cleavage of effector caspase-3 and PARP-1 was analysed by immunoblot. GAPDH was used as a gel-loading control. **d** MDA-MB-231 cells were treated for 8 h with 5 µg/mL C#2, C#11, C#22 or C#24 in the presence or absence of 1 µg/mL His-TRAIL and apoptosis (upper part) or caspase activation (lower part) were analysed as above by flow cytometry or immunoblot. **e** HCT116-DKO cells reconstituted with only DR5 (HCT116 DKO-DR5^rec^) were treated with 10 µg/mL of C#21 or C#23 in the presence or absence of 1 µg/mL His-TRAIL and apoptosis was quantified by flow cytometry. Significance was evaluated by ANOVA test with the mean values ± SD (n = 3) *p < 0.1, **p < 0.05

The use of recombinant TRAIL or agonist antibodies targeting DR4 or DR5 alone to treat patients suffering from cancer is unlikely to translate to the clinic due to the poor ability of these agents to trigger sufficient apoptosis alone^3,4^. However, increasing evidence suggest that it may be possible to combine TRAIL with antibodies targeting DR4 or DR5 to induce efficient apoptosis^13,43–45^. Indeed, several of the antibodies that we have produced here in this study are able to enhance TRAIL-induced apoptosis. Accordingly, and as assessed by methylene blue, combined treatments in HCT116 and MDA-MB-231 cells lines with varying concentration of TRAIL and mAbs for 16 h revealed that 4 out of the 9 mAbs were able to synergize with TRAIL to inhibit the cell viability of these tumour cell lines (Fig. 4). More specifically, albeit not observed in the HCT116 cell line, two anti-DR4 (C#2 and C#11) and two anti-DR5 mAbs (C#22 and C#24) potentiated apoptosis induced by TRAIL in the resistant MDA-MB-231 cell line (Fig. 4b). Their synergistic potential was also clearly evidenced in the BL2 cell line, as monitored by Annexin V staining (Fig. 4c, to compare with Fig. 2a). In these cells, apoptosis induced by TRAIL combined to the selected DR4 or DR5 antibodies was fully abrogated by Q-VD-Oph, as assessed by flow cytometry (Fig. 4c). Consistent with these findings, cleavage of the effector caspase-3 and PARP as well as appearance of PARP cleaved products, in these cells, was enhanced by the combination as compared to cells stimulated with TRAIL or the antibodies alone (Fig. 4c). TRAIL-induced apoptosis-sensitizing potential of C#2, C#11, C#22 and C#24 was also confirmed in MDA-MB-231 cells by Annexin V staining (Fig. 4d). On the other hand, our screen also identified three antibodies displaying inhibitory activity as well, namely C#5, C#21 and C#23. Likewise, although their inhibitory potential appeared modest based on the methylene blue assay in both HCT116 and MDA-MB-231 cells (Fig. 4a, b), and despite the fact that C#5 clearly displayed inhibitory potential in BL2 cells (Fig. 4c), in isogenic cell lines expressing either DR4 (Figure S3A) or DR5 (Fig. 4e), all three antibodies were found to be potent antagonists.

### Characterization of the antibody-binding characteristics by biolayer interference (BLI)

**Fig. 5:**
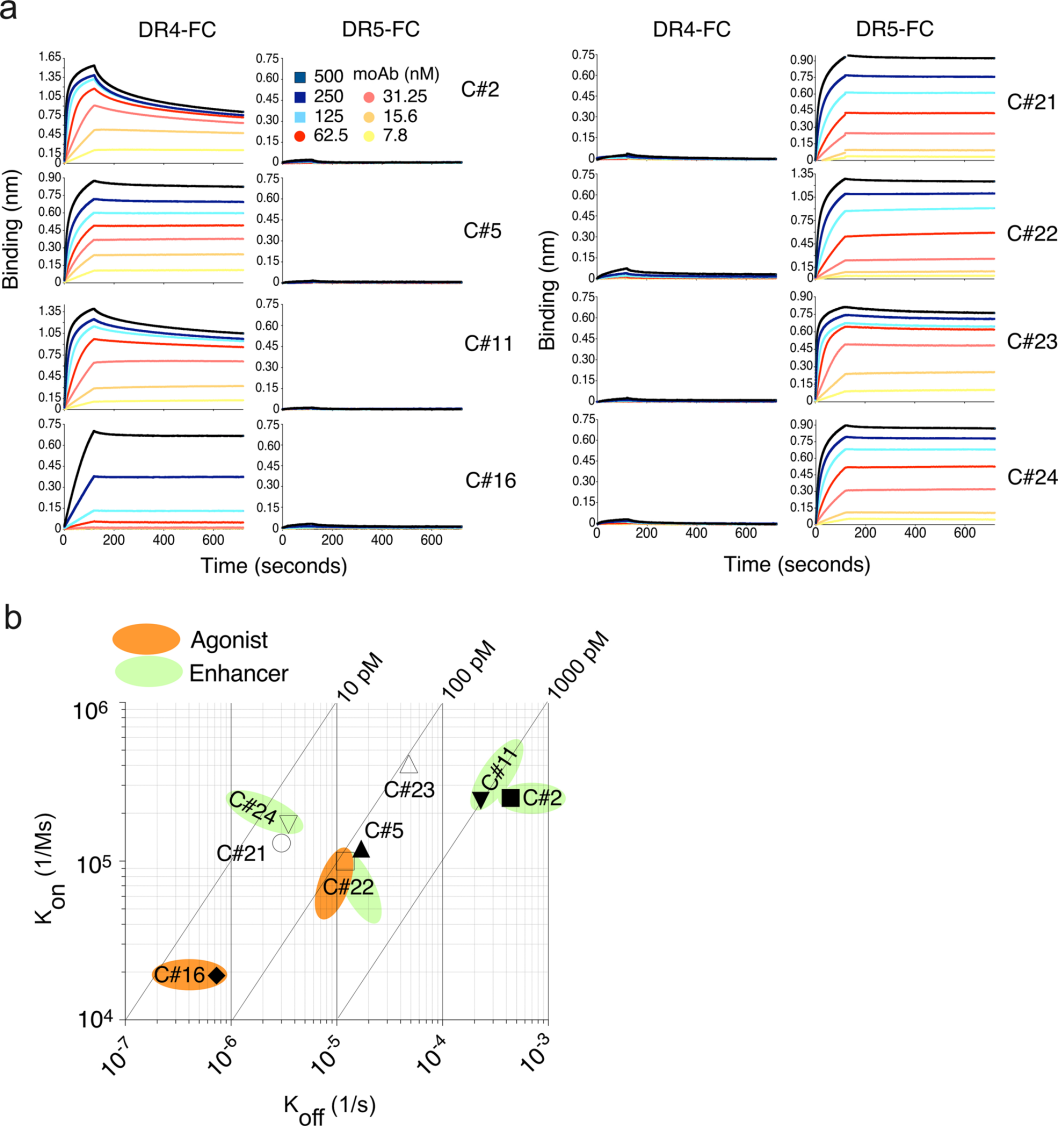
Affinity evaluation of monoclonal antibodies (mAbs). **a** The binding profile of mAbs towards DR4-Fc and DR5-Fc was determined by biolayer interferometry assay. The coloured lines represent the binding response signal at various mAb concentrations (from 500 to 7.8 nM) against DR4-Fc or DR5-Fc. Binding of mAbs was monitored in real time to obtain on (Kon) and off (Koff) rates. Association was measured for 120 s while dissociation was measured for 600 s. The equilibrium constant (KD) was calculated as Koff/Kon using 1:1 binding stoichiometric model. **b** On–off rate map indicating the binding kinetic parameters of anti-DR4 and anti-DR5 antibodies. The association rate constant (Kon) is plotted against the dissociation rate constant (Koff). The diagonal lines indicate the equilibrium dissociation constant (KD). Agonists antibodies (C#16 and C#22) are shown in orange and enhancer antibodies (C#2, C#11, C#22 and C#24) are shown in green

In order to assess whether it may be possible to predict the biological activity of the main DR4 and DR5 antibodies solely based on their binding characteristics and define their affinity for their target, BLI was performed using DR4-Fc and DR5-Fc. The experimental curves were fitted using a 1:1 binding model to calculate the association and dissociation constant rates and KD. All tested antibodies bound specifically to their corresponding receptor in a dose-dependent manner (Fig. 5a), and irrespective of whether they target DR4 or DR5, they displayed high affinity with KDs ranging from 20 pM to ~2 nM (Fig. 5b). Despite the fact that the affinity of some of the generated antibodies is stronger than TRAIL affinity for its cognate receptors^29^, such as C#16 and C#24, only three of them exhibited potent inhibitory activity, suggesting that their binding likely involves distinct amino acids as compared to TRAIL. The anti-DR4 antibody C#5 and the anti-DR5 antibodies (clones C#21 and C#23), on the other hand, are likely to recognize amino acids required for TRAIL binding to DR4 or DR5, respectively, since they all inhibited TRAIL-induced cell death (Fig. 4e and Supplementary Fig. 3A). The biological activity (agonist vs. enhancer) of the remaining antibodies could, however, not be predicted solely based on their binding constants. Likewise, albeit the agonist antibody C#16 displayed the lowest association and dissociation rates among all of the antibodies analysed in this study (Table 1), as illustrated with the on–off rate map (Fig. 5b), neither the association and dissociation constants nor overall affinity for a specific target could be associated with specific biological activity of DR4 or DR5 targeting antibodies. For instance, antibodies exhibiting TRAIL-induced apoptosis-enhancing activity (defined as enhancers mAbs) were all found to display distinct affinity for their target, ranging from 20 to 1740 pM (Table 1 and Fig. 5b). The biological properties of these antibodies are thus more likely due to specific recognition of the target in its native form.

**Table 1:** Kinetic parameters of DR4 and DR5 mAbs towards DR4-Fc and DR5-Fc using BLI

		Enhancers						
	Agonists					Antagonists		
	C#16	C#22	C#2	C#11	C#24	C#5	C#21	C#23
KD	3.8E−11	1.01E−10	1.74E−09	9.32E−10	2.05E−11	1.3E−10	2.3E−11	1.18E−10
Kon	1.9E+04	1E+05	2.5E+05	1.2E+05	1.7E+05	2.4E+05	1.3E+05	4.1E+05
Koff	7.3E−07	1.2E−06	4.4E−04	2.3E−06	3.5E−06	2.2E−04	3E−06	4.8E−05
X2	0.035	0.507	1.12	0.629	0.204	0.637	0.116	0.223
R2	0.99997	0.99934	0.99216	0.99644	0.99903	0.99872	0.99928	0.99896

Equilibrium binding constants (KD) and association (kon) and dissociation (koff) rates were determined using biolayer interferometry (BLI) using 1:1 binding kinetics. Bindings of agonistic antibodies (C#16 and C#22), enhancers antibodies (C#2, C#11, C#22 and C#24) and antagonists (C#5, C#21 and C#23) to soluble recombinant versions of DR4 (DR4-Fc) or DR5 (DR5-Fc) fused to the constant fraction of human gamma-immunoglobulin were evaluated

Altogether, our work provides strong evidence that DNA immunization afforded generation of novel anti-DR4 and anti-DR5 antibodies recognizing native epitopes and displaying potential therapeutic effects.

## Discussion

Given that cancer cells generally overexpress DR4 and DR5^46–49^ and that, for a reason that still needs to be defined, signal transduction of apoptosis induced by these receptors appears to be fairly selective for tumour cells^1,8,50^, targeting TRAIL agonist receptors remains an interesting strategy in current anticancer drug development^51^. Because these receptors represent easy to reach targets, a number of TRAIL derivatives, including recombinant TRAIL, peptidomimetics and agonistic mAbs targeting TRAIL receptors, have been generated and found to be able induce apoptosis in tumour cells in preclinical models^4,50^. However, whereas TRAIL or its derivatives, including anti-DR4 or anti-DR5 mAbs, have been reported to be safe in all animal models and well tolerated in humans, their clinical efficacy has, so far, proven to be rather limited^3,4,52^.

Development of additional anti-DR4 and anti-DR5 mAbs exhibiting potent proapoptotic or TRAIL sensitizing activity potential could nonetheless be interesting to cure patients suffering from cancer. To achieve such an aim, we chose the hydrodynamic genetic immunization method that, to our knowledge, has never been used to generate anti-DR4 or anti-DR5 antibodies. This approach allowed us to generate monoclonal anti-DR4 and anti-DR5 antibodies displaying high affinity and specificity for their target, respectively, with high titres. Generated antibodies were not only almost all directed against the native conformational forms of DR4 or DR5 but also a high proportion of them displayed therapeutic potential, ranging from agonistic, TRAIL-enhancing to TRAIL-inhibiting activities.

Most TRAIL receptor-targeting agonist antibodies require cross-linking or immobilization of protein A or G^53,54^ or immunoglobulin class switch^14^ to unveil their proapoptotic potential^4^. Indeed, out of the 21 mAbs generated in this study, 5 anti-DR4 antibodies required cross-linking to trigger apoptosis (Figure S3B). Remarkably, however, the anti-DR4 C#16 or the anti-DR5 C#22 mAbs were able to induce cell death in HCT116 and BL2 in the absence of cross-linking. Unlike C#22 (not shown), but like Mapatumumab, albeit to a lesser extent, the proapoptotic activity of C#16 could still be increased after antibody-mediated cross-linking (Figure S3C). It should be stressed here that, while the cross-linking efficacy cannot be compared between our antibodies and Mapatumumab due to the use of species-specific cross-linking antibodies, this approach enabled us to unmask the proapoptotic potential of additional anti-DR4 mAbs including C#8, C#13 or C#14, further demonstrating that genetic immunization could be a method of choice to generate antibodies displaying therapeutic properties. These results are particularly remarkable given the limited number of hybridomas screened in our study.

In the breast carcinoma cell line MDA-MB-231, however, the agonistic antibodies C#16 and C#22 were, alone, less efficient, owing to the fact that this cell line is naturally resistant to TRAIL-induced apoptosis due to high expression levels of the caspase-8 inhibitor cFLIP^34^. It should be added here that Mapatumumab, the only anti-DR4 assessed in the clinic, albeit as efficient as C#16 in inducing apoptosis in the BL2 cells, was also found previously to be poorly efficient in triggering apoptosis in MDA-MB-231 cells^12^.

However, and consistent with recent findings highlighting the ability of anti-DR4 and anti-DR5 antibodies to increase TRAIL-apoptosis signalling^13,14,43^, the anti-DR5 C#22 mAb was found to be able to enhance apoptosis induced by TRAIL in the resistant MDA-MB-231 cell line^34^. Importantly, besides C#22, another anti-DR5 antibody C#24, as well as two anti-DR4 antibodies, C#2 and C#11, were also found to be effective in enhancing apoptosis, both in the sensitive BL2 and the resistant MDA-MB-231 cells, when combined with TRAIL.

While it will be required to determine how these antibodies cooperate with TRAIL to increase apoptosis, the synergistic potential appears to be strictly related to the targeted receptor. Likewise, and as demonstrated here using isogenic cells expressing either DR4 or DR5^12^, even in the absence of the non-targeted receptor, combined treatments elicited stronger apoptosis than the addition of the single agents (Figure S3D).

All of the produced anti-DR4 and anti-DR5 antibodies bound to DR4 or DR5, respectively, and recognized their target in a conformational manner. Consistent with a recent study showing that the antitumour effect of DR4 agonist antibodies was not correlated with affinity, per se, but with epitope recognition^14^, the kinetic parameters of the different antibodies produced and analysed in our study were not predictive of their biological properties, suggesting that they might recognize different epitopes. Henceforth, while all our antibodies bound their target with high affinity, ranging from 20 pM to 2 nM, and albeit their association constants were fairly similar, with the exception of C#16, their dissociation characteristics differed substantially from 7 × 10^−7^ to 4 × 10^−4^ (s^−1^). Whereas clone C#16 bound more slowly to its target, its overall affinity was high due to its low dissociation rate. On the other hand, DR4 enhancers (C#2 and C#11) both associated and dissociated very rapidly to and from their target while displaying a 100-fold lower affinity as compared to C#16, respectively. On the other hand, the kinetic parameters of the DR5 enhancers, C#24 and C#22, were clearly dissimilar. With a lower association constant rate as well as a lower dissociation rate, these enhancers displayed a 10–100-fold increase in affinity as compared to DR4 enhancers. Although it remains to be determined whether C#2 and C#11 may be able to bind to the same epitope, due to their affinity constant, C#24 and C#22 are unlikely to share the same epitope since C#22, contrary to C#24, displays both agonistic and synergistic properties. Taken together, these results substantiate the finding that TRAIL-mediated cell death can significantly be enhanced both by anti-DR4 and -DR5 mAbs.

Humanization and/or engineering optimization of the best leads will be required before considering these novel murine anti-DR4 and -DR5 antibodies for cancer therapy, including clone C#16. In particular, after humanization it will be important to determine whether the antitumour growth effects found in our study with this clone is solely mediated through its ability to trigger apoptosis through DR4 or whether its antitumour potential may also involve, at least partially, antibody-dependent cell-mediated cytotoxicity (ADCC) and/or complement-dependent cytotoxicity. In line with ADCC, several of the anti-DR4 or -DR5 antibodies raised here, irrespective of their ability to trigger apoptosis in the absence of cross-linking or combined with TRAIL, could potentially be of interest to deliver toxic agents to the tumour site.

## Conclusion

Genetic immunization, unlike conventional approaches, is more likely to preserve not only the shape of the protein of interest but also their posttranslational modifications, allowing not only the generation of antibodies recognizing hard-to-produce transmembrane proteins but also antibodies displaying pharmacological properties. To our knowledge, this is the first time that genetic immunization has been used to generate antibodies against death receptors. Anti-DR4 and anti-DR5 mAbs generated using this DNA approach, as demonstrated here not only displayed, as expected, high affinity to their native targets, but remarkably, among the limited number of purified clones, almost 12 out of 21 displayed pharmacological properties. Beyond TRAIL, this proof of concept clearly opens novel opportunities for the use of genetic immunization to generate potential therapeutic antibodies.

## Supplementary information

Supplementary text

Supplementary Figure 1

Supplementary Figure 2

Supplementary Figure 3
