# Supplementary material for: Generation and characterization of novel anti-DR4 and anti-DR5 antibodies developed by genetic immunization
Source: Cell Death Dis. 2019 Feb 4;10(2):101. doi: 10.1038/s41419-019-1343-5 (PMC6362131; doi:10.1038/s41419-019-1343-5)
Supplement: Supplementary file 1 — Supplementary text [file 41419_2019_1343_MOESM1_ESM.docx]

Supplementary material

**Supplementary materials and methods**

For cross-linking assays, rabbit anti-mouse and mouse anti-human secondary antibodies from Jackson ImmunoResearch (Interchim, Montluçon, France) were used. The inhibitory effect of C#5 was screened on MDA-MB-231-DKO-DR4^rec^ cells. Cells were plated at 5x10^5^ cells/mL in 24-well plate and incubated with C#5 at 10 µg/mL alone or combined to 1 µg/mL His-TRAIL for 20 h. Supernatants and cells were collected and apoptosis was quantified by allophycocyanin Annexin V / 7-AAD staining, according to the manufacturer’s instructions (BD Biosciences), and analysed by flow cytometry (FACS LSRII). Cross-linking experiments were performed on BL2 cells. Cells were plated at 2x10^5^ cells/mL in 24-well plate and incubated with mAbs at 5 µg/mL in the presence or absence of 5 µg/mL anti-mouse or anti-human cross-linking antibody for 20 h. Supernatants and cells were collected and apoptosis was quantified as described above.

**Supplementary Figure 1:** **Screening of hybridoma supernatants and monoclonal antibodies (a)** Hybridoma supernatants were diluted by half and tested on recombinant cleaved soluble receptors, DR4 (cDR4) or DR5 (cDR5), Fc or BSA. **(b-c)** Supernatants of selected anti-DR4 or DR5 hybridomas were screened by flow cytometry on parental HCT116 cells **(b)** or MBA-MB-231 cells deficient for both DR4 and DR5 (DKO) and MBA-MB-23-DKO reconstituted for DR5 **(c)**, respectively. Specific mAbs staining (light red curves) were compared to two commercially available antibodies detecting DR4 or DR5 (red and blue curves, respectively) and to a non-relevant control IgG1 isotype antibody (grey curve).

**Supplementary Figure 2:** **Characterisation of produced anti-DR4 and anti-DR5 mAbs (a)** Evaluation of purified antibody titres by ELISA. Purified antibodies were diluted by half, starting from an initial concentration of 500 ng/mL, on recombinant cDR4, cDR5, Fc or BSA and titres were defined as the highest dilution giving an optical density equal or above 1. **(b)** Recognition of non-denaturated (left) and denaturated forms (right) of recombinant cDR4 (above) and cDR5 (below) by mAbs was evaluated by dot blot. cDR4 and cDR5 (150 ng/µl) were denaturated using 10% of 1 M DTT (dithiothreitol) and heat (95°C for 5 min) and deposited onto nitrocellulose membranes. After blocking with milk, dot blots were incubated with 1 µg/mL purified mAb, and binding of primary antibody was revealed using a secondary anti-mouse IgG (H+L) antibody coupled to the HRP followed chemoluminescence. **(c)** Schematic representation of the isotypic landscape of the DR4 and DR5 mAbs generated in this study.

**Supplementary Figure 3**: **(a)** Inhibitory potential of the anti-DR4 mAb C#5. MDA-MB-231-DKO reconstituted with DR4 (MDA-MB-231-DKO-DR4^rec^) cells were treated with 10 µg/mL of C#5, His-TRAIL (1 µg/ml) or the combination of both compounds for 20 h and apoptosis was quantified by flow cytometry. Significance was evaluated by ANOVA test with the mean values +/- SD (n=3) ** p < 0.05. **(b)** Apoptotic potential of cross-linked anti-DR4 mAbs. BL2 cells were treated for 20 h with 5 µg/mL of mAbs alone (white) or mAbs cross-linked with 5 µg/mL of secondary anti-mouse antibody (grey). Cells were harvested and apoptosis was detected via Annexin V / 7AAD binding by flow cytometry. **(c)** 1 µg/mL of C#16 and Mapatumumab were tested alone (white) or with 1 µg/mL of a secondary anti-mouse or anti-human antibody (grey), respectively, on BL2 cells for 20 h. Cross-link effect on the induction of apoptosis was evaluated by flow cytometry as described above. **(d)** The synergic potential of enhancers antibodies was assessed using HCT116 parental (white bars) or isogenic cells expressing either DR4 (blue bars) or DR5 (red bars). Cells were stimulated for 20h with the anti-DR4 enhancer antibodies mAbs C#2 and C#11, anti-DR5 antibodies mAbs C#22 and C#24, His-TRAIL or with the combination, as described figure 4C, and apoptosis was evaluated by annexin V staining.
