# Supplementary figures and images for: Generation and characterization of novel anti-DR4 and anti-DR5 antibodies developed by genetic immunization
Source: Cell Death Dis. 2019 Feb 4;10(2):101. doi: 10.1038/s41419-019-1343-5 (PMC6362131; doi:10.1038/s41419-019-1343-5)

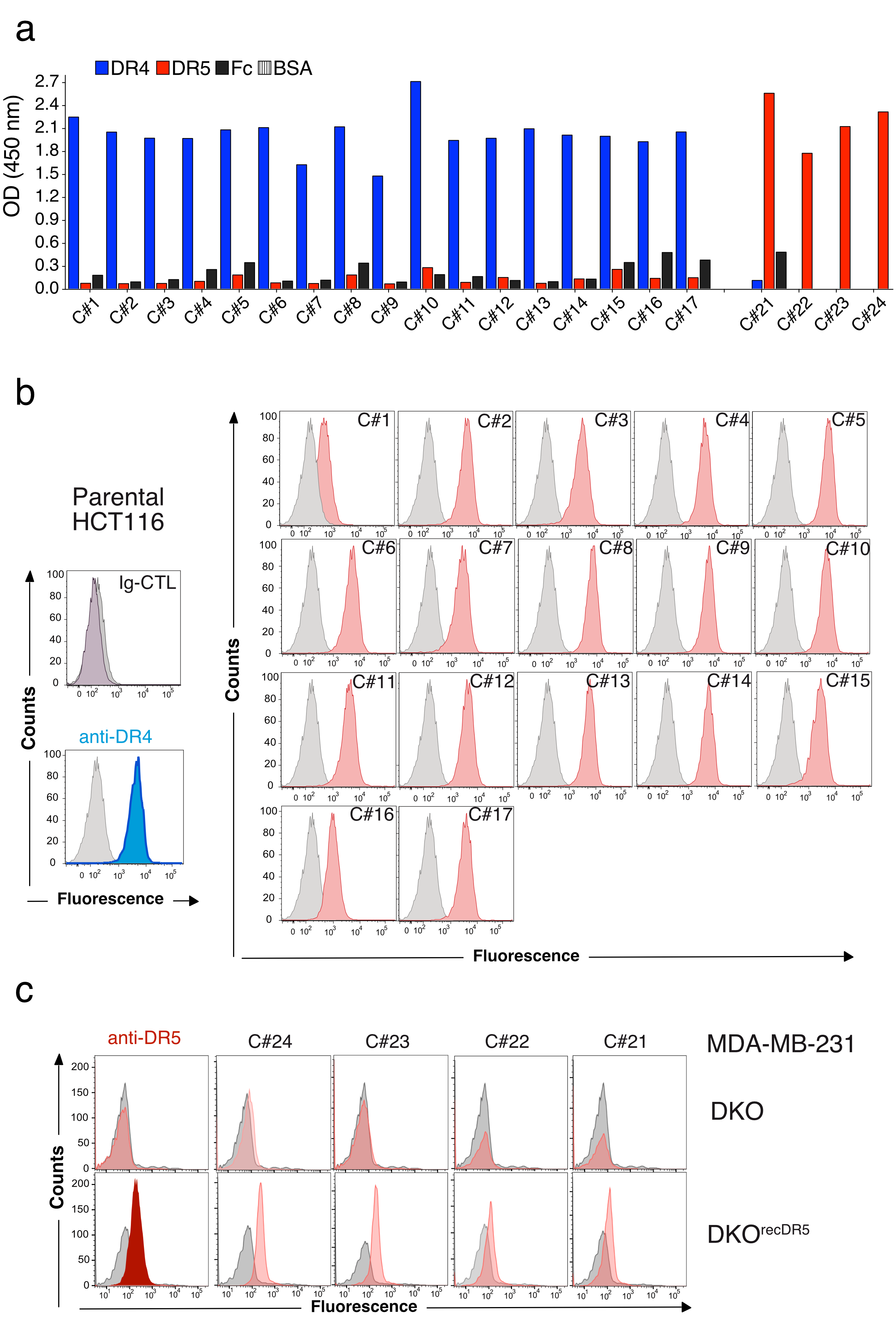

Supplement: Supplementary file 2 — Supplementary Figure 1 [file 41419_2019_1343_MOESM2_ESM.tiff]

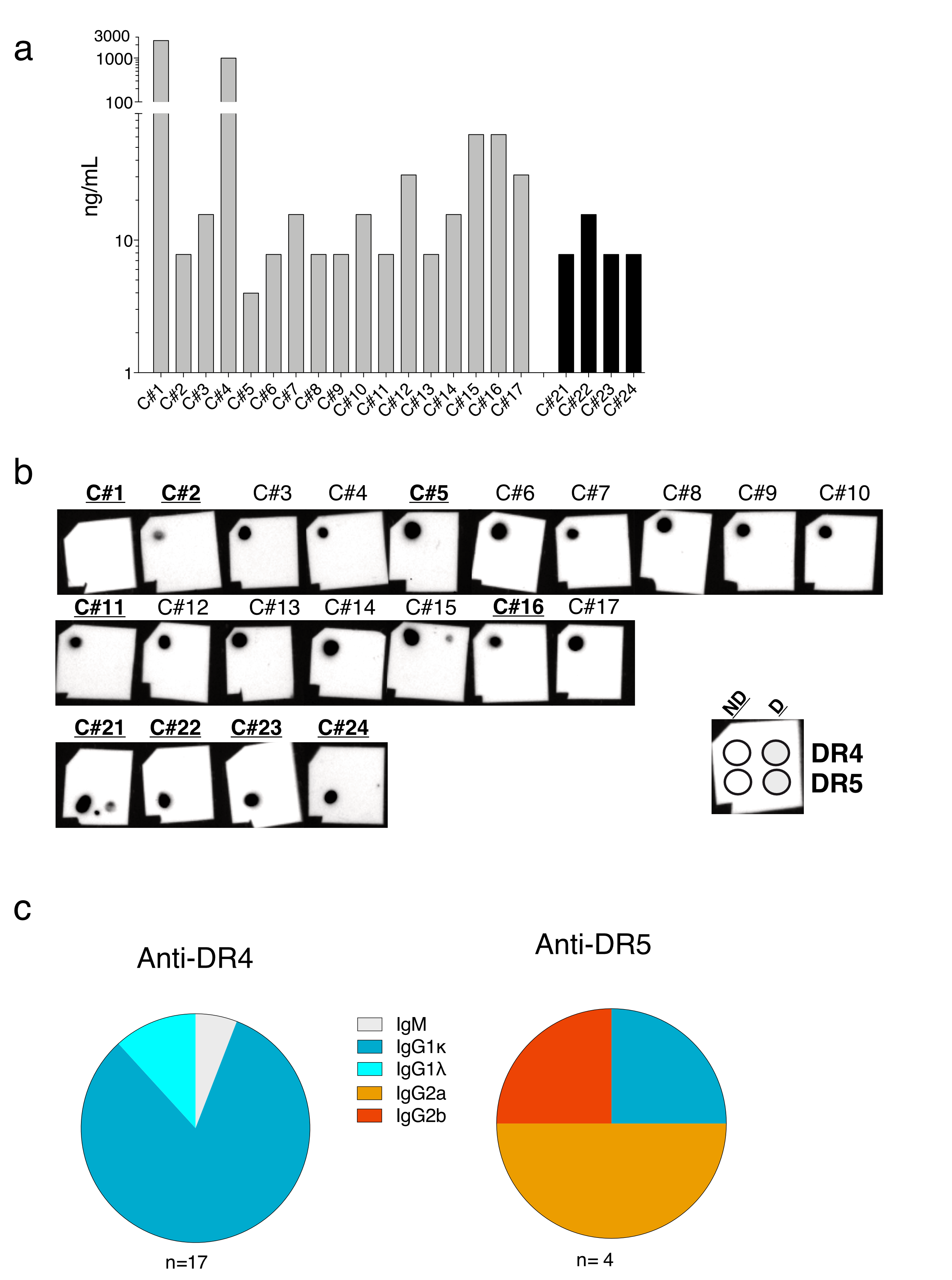

Supplement: Supplementary file 3 — Supplementary Figure 2 [file 41419_2019_1343_MOESM3_ESM.tiff]

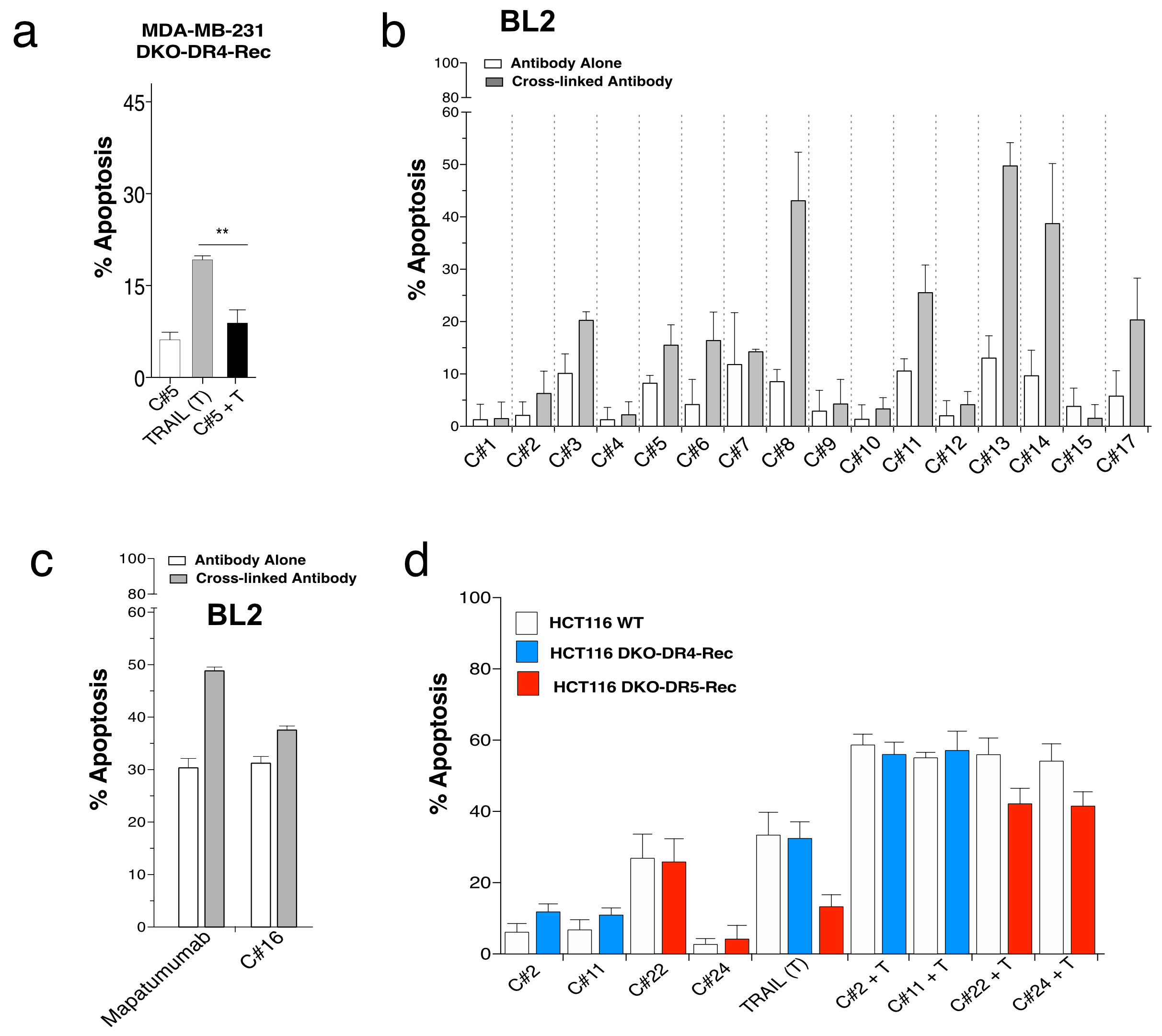

Supplement: Supplementary file 4 — Supplementary Figure 3 [file 41419_2019_1343_MOESM4_ESM.tiff]
